# Response to the Commentary „Can a 1-Item Scale for Psychotherapy Outcomes Be Psychometrically Robust?”

**DOI:** 10.32872/cpe.16921

**Published:** 2025-02-28

**Authors:** Brian Schwartz, Miguel M. Gonçalves, Wolfgang Lutz, João Tiago Oliveira, Suoma E. Saarni, Orya Tishby, Michael Barkham, European Psychotherapy Consortium (EPoC)

**Affiliations:** 1 Department of Psychology, Trier University, Trier, Germany; 2 CIPsi – Psychology Research Center, School of Psychology, University of Minho, Braga, Portugal; 3 Department of Psychiatry, Faculty of Medicine and Health Technology, Tampere University, Tampere, Finland; 4 Department of Psychiatry, Helsinki University Hospital HUS and Helsinki University, Helsinki, Finland; 5 Department of Psychiatry, Wellbeing Services County of Päijät-Häme, Lahti, Finland; 6 Department of Psychology, Hebrew University, Jerusalem, Israel; 7 Clinical and Applied Psychology Unit, School of Psychology, University of Sheffield, Sheffield, United Kingdom

We thank the author(s) for their commentary in response to our article on the European Psychotherapy Consortium (EPoC) of the European Chapter of the Society for Psychotherapy Research (SPR) published in *Clinical Psychology in Europe* (Gonçalves et al., 2024) and welcome the opportunity to respond. Whilst we appreciate the detailed comments on our article made by the author(s), we would like to take this opportunity to primarily address the specific criticisms of the EPO-1 single-item scale.

Overall, the commentary criticizes our article on a combined theoretical and psychometric basis as a first, but so far, successful, attempt to coordinate the administration of patient outcome data collected during the course of treatment and implemented across Europe. Such criticism fails to recognize the practical impact of our strategic approach, which aims to significantly advance the paradigm of patient-focused research. Our objective has been to advance the field of psychological therapies and pave the way for the first steps towards coordinated data collection, data-based quality assurance, and practice-based evidence into the therapeutic process across national borders. The single-item Emotional and Psychological Outcome-1 measure (EPO-1) addresses the reality of identifying a common measurement language across different clinics in different European countries with varying structures of assessment and other healthcare systems, some with already existing measurement systems and some with none. Hence, we are attempting what we consider to be a unique program of research implementation that is both feasible in most clinical settings (i.e., minimal demands on patients, therapists, and healthcare systems) and yet has a grand vision in transcending national boundaries that are, so often, limitations to research collaboration. Against this background, the commentary fails to fully appreciate the objectives and value of the EPO-1 item’s introduction.

In addition to presenting our general view of the project, which is broader than that of the commentary’s author(s), we would also like to respond to some specific points of criticism below.

The author(s) assumes that the EPO-1 item has limited reliability and validity. In response, we direct the author(s) to the high correlations of the instrument with other established outcome instruments, such as the Outcome Questionnaire-30 (OQ-30; *r* = .601), the Questionnaire for the Evaluation of Psychotherapy (FEP-2; *r* = .626), and the Patient Health Questionnaire-9 (PHQ-9; *r* = .630), which can be found in detail in Chapter 4 (Appendix) of *Bergin and Garfield’s handbook of psychotherapy and behavior change* (Lutz et al., 2021). The empirical data (*n* = 521) also show that the pre-post effect sizes, measured with the EPO-1, are as strongly related to the above instruments and that the individual effect sizes are comparable to those of the other measures. The EPO-1 pre-post-effect sizes refute the assumption that the single-item measure EPO-1 is less sensitive to change than multi-item scales (pre-post-effect sizes, e.g., EPO-1(Likert): *d* = 1.086; EPO-1(analogue): *d* = 1.469; BSI: *d* = 0.879; OQ-30: *d* = 1.320; Lutz et al., 2021).

For future research, the author(s) recommends demonstrating the stability of the item over time without intervention, its change in response to intervention, and its correlations with established measures. The last two points (change sensitivity and convergent validity) have already been empirically demonstrated (see above; Lutz et al., 2021), leaving only the first point (reliability in the absence of intervention) to be addressed in future studies. Many points of criticism in the commentary refer to the general disadvantages of self-report questionnaires. They are not specifically related to single-item scales (e.g., the varying interpretation of questions by individual patients or the cognitive complexity of evaluating the item).

Further, the author(s) suggests extending the EPO-1 item with an alliance item and using large language models (LLMs), which are important and topical issues in recent psychotherapy research. However, the EPO-1 is intentionally designed as a *single-item measure* to ensure easy implementation in clinical practice. Moreover, this outcome measure assesses psychological well-being, not therapeutic alliance. While LLMs have become valuable tools in psychotherapy research, the EPO-1 item is a low-burden measure for patients, collected via self-report. Therefore, it should not be replaced by video or text analyses, which could capture a different perspective.

Our response addresses the concerns expressed based on theoretical considerations with empirical evidence. Furthermore, we would argue that the benefits and potential of these efforts to introduce a standardized outcome measure across Europe outweigh the theoretical (and, as demonstrated, not necessarily valid) criticisms. We are aware of the common problems of single-item scales, which is why the EPoC does not only focus on the EPO-1 item but also on developing and implementing crosswalks to create a standard measure structure between different clinics across Europe.

In summary, EPoC is a project that aims to evolve and create large, heterogeneous data sets from different countries that will facilitate practice-based evidence and data-informed psychological therapy. EPO-1 is currently translated into 13 languages, and two more are expected to be added soon. We hope that more institutions will join our initiative in the future and adopt the item in their assessments. Ultimately, whether it is taken up in the field is an empirical question.

## Social Media Accounts

*Brian Schwartz:* Bluesky: @schwartz-psyres.bsky.social
